# Risk Stratification and Optimal Use of Implantable Cardioverter-Defibrillator Therapy in Primary Prevention of Sudden Cardiac Death in Genetic Cardiomyopathies, with Assessment of the Role of Genetic Variants in Guiding Therapeutic Decisions

**DOI:** 10.3390/biomedicines13112626

**Published:** 2025-10-27

**Authors:** Eleonora Ruscio, Roberto Scacciavillani, Filippo Luca Gurgoglione, Gaetano Pinnacchio, Gianluigi Bencardino, Francesco Perna, Maria Lucia Narducci, Gemma Pelargonio, Giampaolo Niccoli, Gabriella Locorotondo, Francesco Burzotta

**Affiliations:** 1 Department of Cardiovascular Sciences, Fondazione Policlinico Universitario Agostino Gemelli IRCCS, 00168 Rome, Italy; roberto.scacciavillani@gmail.com (R.S.); gaetano.pinnacchio@policlinicogemelli.it (G.P.); gianluigi.bencardino@policlinicogemelli.it (G.B.); francesco.perna@policlinicogemelli.it (F.P.); marialucia.narducci@policlinicogemelli.it (M.L.N.); gemma.pelargonio@policlinicogemelli.it (G.P.); gabriella.locorotondo@policlinicogemelli.it (G.L.); francesco.burzotta@policlinicogemelli.it (F.B.); 2 Department of Cardiology, Parma University Hospital, 43126 Parma, Italy; filippolucagurgoglione@gmail.com (F.L.G.); giampaolo.niccoli@unipr.it (G.N.); 3 Cardiology Institute, Catholic University of Sacred Heart, 20123 Rome, Italy

**Keywords:** genetic cardiomyopathies, sudden cardiac death, implantable cardioverter-defibrillator, genotype-based risk stratification, precision medicine

## Abstract

Genetic background is a critical determinant of disease expression, arrhythmic vulnerability, and therapeutic response in inherited cardiomyopathies. Implantable cardioverter-defibrillators (ICD) remain the cornerstone for primary prevention of sudden cardiac death, yet conventional selection based on left ventricular ejection fraction does not adequately reflect the heterogeneity of genetic substrates. Increasing evidence demonstrates that pathogenic variants differ not only in prevalence across cardiomyopathy subtypes but also in prognostic impact. Truncating variants, particularly in genes encoding structural proteins, are often associated with severe remodeling, progressive dysfunction, and high arrhythmic risk, whereas missense variants may confer variable expressivity, ranging from aggressive arrhythmogenic phenotypes to milder or late-onset disease. This variability underscores the importance of distinguishing variant classes in clinical decision-making. Integrating genetic information with advanced imaging markers, such as late gadolinium enhancement, allows refinement of arrhythmic risk stratification beyond static thresholds and supports more tailored ICD allocation. Nevertheless, translation into routine practice is limited by challenges in variant interpretation, phenotypic overlap between cardiomyopathy subtypes, and the lack of prospective validation of genotype-based models. In the precision medicine era, evolving strategies should move toward dynamic, multimodal approaches that combine genotype, phenotype, and imaging biomarkers, enabling more accurate prediction of arrhythmic risk and more cost-effective use of ICD therapy.

## 1. Introduction

Genetic predisposition is increasingly recognized as a key determinant of risk stratification and response to implantable cardioverter-defibrillator (ICD) therapy in primary prevention of sudden cardiac death (SCD) [1]. Many life-threatening ventricular arrhythmias (VAs) and SCD, especially in the young, can be attributed to an expanding spectrum of genetically mediated arrhythmogenic disorders [2,3]. In cardiomyopathies, arrhythmogenicity is largely linked to structural substrates such as hypertrophy, fibrosis, and fibro-fatty replacement [4,5], while in channelopathies it reflects ion channel dysfunction [6]. Increasing evidence highlights a complex interplay between structural and electrophysiological abnormalities in the genesis of malignant arrhythmias [7,8].

The ICD is the most effective therapy for preventing SCD [3], improving survival particularly in young patients with inherited cardiomyopathies [9]. However, device-related complications, including inappropriate shocks and lead issues, necessitate careful patient selection [10]. Subcutaneous ICD provide an alternative to transvenous systems, though with limitations such as lack of pacing [11,12]. While ICD is established for secondary prevention, its role in primary prevention is still under investigation [2,3].

Advances in genomic medicine underscore the importance of pathogenic/likely pathogenic (P/LP) variants and variants of uncertain significance (VUS) in refining individualized ICD strategies [13]. Detailed variant characterization, including protein-level changes, pathogenicity classification, and quantitative score, may improve arrhythmic risk models and guide ICD eligibility decisions. Integrating genetic data into clinical practice could therefore enhance predictive accuracy and optimize the balance between arrhythmic protection and device-related morbidity [2].

This review discusses risk stratification and the optimal use of ICD therapy in primary prevention of SCD in genetic cardiomyopathies, with particular focus on the role of genetic variants in guiding therapeutic decisions.

## 2. Cardiomyopathies: Clinical Phenotypes and Genetic Determinants

Cardiomyopathies comprise a heterogeneous group of phenotypes, hypertrophic, dilated, arrhythmogenic, and restrictive, traditionally defined by structural and functional features [1,2,3]. While conventional imaging and clinical assessment remain central, genetic profiling now plays a critical role in prognostication and personalized management. Current guidelines underscore the importance of genotype-informed risk stratification across inherited cardiomyopathies [2,3]. Mechanistic insights show that genetic variants disrupt sarcomeric function, intercellular adhesion, or nuclear envelope integrity, directly linking molecular defects to phenotype and arrhythmic vulnerability [14].

### 2.1. Dilated Cardiomyopathy and Non-Dilated Left Ventricular Cardiomyopathy

Dilated cardiomyopathy (DCM) is defined by left ventricular (LV) dilatation with systolic dysfunction, in the absence of abnormal loading or significant coronary artery disease [2]. Etiology may be genetic or acquired, with environmental modifiers such as peripartum state, alcohol, or chemotherapy [2]. Genetic DCM can evolve from early phenotypes, including hypokinetic non-dilated cardiomyopathy (HNDCM), within the broader non-dilated LV cardiomyopathy (NDLVC) spectrum, often identified by non-ischemic scarring or fatty replacement on cardiac magnetic resonance [2].

P/LP variants are identified in approximately 35–40% of DCM and up to 55% of familial cases, usually autosomal dominant [2]. Frequently implicated are cytoskeletal and regulatory genes, including lamin A/C (LMNA), transmembrane protein 43 (TMEM43), desmoplakin (DSP), phospholamban (PLN), filamin C (FLNC), and RNA-binding motif protein 20 (RBM20). Sarcomeric contributors include truncating variants in titin (TTN), the most prevalent genetic cause (20–25% of unselected cohorts), as well as actin alpha cardiac muscle 1 (ACTC1), actinin alpha 2 (ACTN2), tropomyosin 1 (TPM1), troponin T type 2 cardiac (TNNT2), and troponin I type 3 cardiac (TNNI3) [2]. Additional loci underscore mechanistic diversity, such as BCL2-associated athanogene 3 (BAG3), a co-chaperon highlighting cytoskeletal disruption, and sodium voltage-gated channel alpha subunit 5 (SCN5A), linking ion channel dysfunction to DCM even without syndromic features [15]. Mitochondrial and metabolic pathways also contribute, exemplified by tafazzin (TAZ) in Barth syndrome [16]. Genotype-positive patients, particularly those with nuclear envelope, desmosomal, or cytoskeletal variants, show worse outcomes [17].

### 2.2. Hypertrophic Cardiomyopathy

Hypertrophic cardiomyopathy (HCM) is characterized by increased LV wall thickness or mass not attributable to abnormal loading, sometimes involving the right ventricle (RV) [2]. It is most frequently due to autosomal dominant sarcomeric mutations, particularly in β-myosin heavy chain (MYH7) and cardiac myosin-binding protein C (MYBPC3), identified in 30–60% of cases, especially in younger individuals and familial clusters [4]. SCD predominantly affects younger patients, mediated by VAs, ischemia, or LV outflow tract obstruction (LVOTO), while older patients more often succumb to heart failure or stroke [2]. Although once restricted, vigorous physical activity is now considered safe in the absence of conventional risk markers [18].

### 2.3. Arrhythmogenic Cardiomyopathy

Arrhythmogenic cardiomyopathy (ACM) is characterized by progressive myocardial atrophy with fibro-fatty replacement, predisposing to malignant VAs [2]. Initially recognized as arrhythmogenic RV cardiomyopathy, diagnostic frameworks have evolved to improve sensitivity and specificity and now acknowledge LV forms and phenocopies, such as myocarditis or sarcoidosis, which differ in prognosis and management [19].

P/LP variants are most frequently identified in desmosomal genes, including plakophilin 2 (PKP2), DSP, desmoglein 2 (DSG2), desmocollin 2 (DSC2), and junction plakoglobin (JUP). Non-desmosomal contributors include desmin (DES), TMEM43, and PLN [20,21]. Variants are detected in up to 60% of cases, usually autosomal dominant, with penetrance influenced by age, sex, and exercise [2].

### 2.4. Restrictive Cardiomyopathy

Restrictive cardiomyopathy (RCM) is defined by restrictive hemodynamics with diastolic dysfunction, atrial enlargement, and non-dilated ventricles, irrespective of wall thickness or systolic performance [22]. Restrictive filling patterns generally appear late, though milder dysfunction is often present at diagnosis. Careful phenotyping, including extracardiac features, is essential, particularly in syndromic or neuromuscular contexts. According to the Rapezzi framework, RCM encompasses interstitial fibrosis, extracellular infiltration, intracellular storage, and endomyocardial fibrosis, distinguishing genetic from acquired causes such as amyloidosis or endomyocardial disease [22].

Genetic RCM is usually autosomal dominant, though recessive and sporadic cases occur. Sarcomeric mutations predominate, involving TNNI3, TNNT2, ACTC1, MYH7, and MYBPC3, with additional contributions from titin (TTN), tropomyosin 1 (TPM1), myopalladin (MYPN), and myosin light chains (MYL2, MYL3) [2,22]. Non-sarcomeric variants such as DES, FLNC, and BAG3 promote protein misfolding or aggregation, conferring adverse prognosis, as exemplified by BAG3-related myofibrillar myopathy [23]. Overlap with DCM and HCM underscores variable expressivity of the genetic substrate, complicating risk stratification, while acquired infiltrative, inflammatory, or fibrotic forms emphasize the heterogeneity of the restrictive phenotype [22].

## 3. Mechanisms of Arrhythmogenesis in Cardiomyopathies

Arrhythmogenesis in cardiomyopathies reflects the convergence of structural remodeling, electrophysiological derangements, and genetic determinants [24]. In inherited forms, atrial and VAs are closely linked to myocardial fibrosis, conduction abnormalities, and ion-channel dysregulation, with late gadolinium enhancement (LGE) on CMR serving as a marker of fibrotic substrate (e.g., in LMNA-, FLNC-, and DSP-related disease) [24]. Desmosomal variants, exemplified by loss-of-function mutations in PKP-2, promote arrhythmogenesis not only through impaired intercellular adhesion but also by perturbing intercalated disk architecture. In vitro evidence demonstrates reduced voltage-gated sodium channel (NaV1.5) localization, diminished sodium current density, and slowed conduction velocity, generating spatial heterogeneity of depolarization and a substrate highly susceptible to re-entrant VAs [25]. Additional sodium-channel dysfunction, neurohormonal activation, and altered calcium homeostasis further contribute to electrical instability [26]. In arrhythmia-induced cardiomyopathy, sustained tachyarrhythmias impose metabolic stress and maladaptive remodeling, leading to chamber dilation and persistent pro-arrhythmic changes, even after rhythm correction [27].

## 4. Non-Genetic Risk Stratification in Cardiomyopathy: The Role of CMR-Detected Myocardial Fibrosis

Myocardial fibrosis is nowadays widely recognized as independent adverse prognostic factor, as it contributes to structural and electrical changes, thus leading to a higher risk of VAs, heart failure and SCD [28,29]. Cardiovascular magnetic resonance (CMR) has emerged as the non-invasive gold standard imaging technique to assess myocardial fibrosis, and the only one non-invasive imaging tool widely validated against histopathological examination [30]. The most used technique to assess replacement myocardial fibrosis is LGE, which is acquired 10–20 min after the injection of gadolinium-based contrast agents. The expansion of extracellular space, due to cardiomyocyte death and fibrotic tissue, retains gadolinium, which leads fibrotic tissue to shine with higher signal intensity compared with the surrounding normal myocardium. Presence and extent of myocardial fibrosis or scar at LGE imaging predict cardiovascular death and all-cause mortality in both ischemic and non-ischemic cardiomyopathies [30].

## 5. Evolving Risk Stratification: Genetic Modulation and Its Integration with Conventional Risk Factors

### 5.1. Dilated Cardiomyopathy: Risk Stratification Beyond Ejection Fraction

The paradigm of primary prevention ICD therapy has historically relied on a left ventricular ejection fraction (LVEF) threshold of ≤35%, a criterion supported by the cumulative evidence of randomized trials and meta-analyses [31]. While this approach has provided a practical framework, its discriminatory capacity is limited in DCM, a condition marked by substantial etiological and genetic heterogeneity. Increasingly, molecular genetics has emerged as a key determinant of arrhythmic risk stratification, informing both prognostic evaluation and therapeutic decision-making [2].

Variant-specific characteristics, encompassing nucleotide- and protein-level changes, pathogenicity classification and integration into validated genotype-based risk models, have assumed clinical relevance. In LMNA-associated disease, truncating variants (nonsense substitution or frameshift) confer a consistently elevated arrhythmic risk irrespective of genomic locus, whereas select missense substitutions, particularly within the tail domain (exons 7–12), remain classified as VUS, being associated with more favorable cardiac outcomes [32,33]. The LMNA-risk VTA score, incorporating truncating variant type alongside sex, LVEF, conduction abnormalities, and non-sustained VT, enables individualized 5-year risk estimation and guides ICD eligibility [34].

The PLN c.40_42delAGA (p.Arg14del) variant, resulting in the in-frame deletion of arginine at codon 14, is a powerful predictor of malignant VAs, even in patients with preserved or moderately reduced LVEF, and a dedicated 5-year risk score has been proposed to refine prognostic assessment [35]. In contrast, non-recurrent missense substitutions or small in-frame indels are typically retained as VUS, reflecting single amino acid changes of PLN without proven consequences for Sarcoplasmic/Endoplasmic Reticulum Calcium ATPase (SERCA2a) regulation or calcium handling [36]. Similarly, truncating variants in FLNC (nonsense substitution or frameshift) are strongly associated with ventricular arrhythmogenesis and adverse remodeling [37,38]. Nonetheless, non-truncating variants in the same gene have also been reported in dilated and mixed cardiomyopathy, with selected substitutions shown to perturb protein structure, alter splicing, or promote aggregation, thereby exerting variable effects on penetrance and clinical outcome [39]. By contrast, truncating TTN variants (including nonsense substitutions, frameshift mutations, splice-site alterations, and occasional large tandem insertions), although the most prevalent cause of DCM, frequently exhibit LVEF recovery and a comparatively attenuated arrhythmic profile under optimized medical therapy [40,41].

BAG3 variants, including the missense BAG3 c.626C>T; p.(Pro209Leu) (proline-to-leucine substitution at codon 209, impairing co-chaperone function), are linked to early-onset DCM, VAs, and progression to advanced heart failure, with ventricular ectopy, LV dilatation, and myocardial fibrosis on CMR emerging as key markers of risk [23]. TAZ loss-of-function variants (frameshift, nonsense, splice site, large deletions/duplications), causative of Barth syndrome, manifest with early DCM, skeletal myopathy, and neutropenia, where severe LV dysfunction, recurrent neutropenia, and defective cardiolipin remodeling predict adverse outcome [16].

Finally, in genotype-negative patients, the presence of LGE on CMR has emerged as an independent and powerful predictor of mortality and malignant VAs, frequently outperforming LVEF as a discriminator of arrhythmic risk [42,43].

### 5.2. Non-Dilated Left Ventricular Cardiomyopathy: Lessons from Genotype and Related Cardiomyopathies

No RCTs have assessed ICD use in patients with mild–moderate LV dysfunction or NDLVC [2]. Current recommendations mirror those for DCM with LVEF < 35%, though most NDLVC patients present with preserved or mildly reduced function.

In this setting, emerging evidence suggests that genotype may help refine arrhythmic risk assessment. FLNC truncating variants are typically associated with NDLVC phenotypes characterized by extensive myocardial fibrosis and a high incidence of VAs and SCD, often irrespective of LVEF [37]. DSP mutations, particularly truncating variants dispersed throughout the gene, may cause left-dominant arrhythmogenic cardiomyopathy with subepicardial LGE, early VAs, and only modest systolic impairment, consistent with a haploinsufficiency mechanism, whereas missense variants cluster within plakophilin/plakoglobin and desmin binding domains, suggesting disruption of critical protein–protein interactions [44]. The PLN p.Arg14del founder mutation predisposes to malignant arrhythmias and progressive heart failure even before overt dilatation, and a dedicated prediction model incorporating electrical and imaging markers outperforms generic DCM/ARVC tools [36]. Missense variants in the arginine–serine–rich domain of RBM20 are linked to a highly aggressive cardiomyopathic phenotype, frequently manifesting with early disease onset, malignant VAs, and a high likelihood of heart transplantation, with a particular predilection in male patients [21]. Experimental data indicate that these variants alter titin splicing and calcium handling, contributing to an arrhythmogenic NDLVC phenotype in which ventricular arrhythmic risk is disproportionate to the degree of systolic dysfunction [45]. Less common associations include DES and TMEM43, both of which may produce NDLVC phenotypes with diffuse fibrosis and arrhythmias despite minimal dilatation [2,20].

In genotype-negative cases, ICD may be considered with NSVT, family history of SCD, or LGE [2]. Other markers (ectopy burden, syncope) and electrophysiological study (EPS) may contribute, but evidence remains limited. ESC 2022 guidelines align NDLVC with DCM, though with lower certainty, emphasizing shared decision-making [2].

### 5.3. Hypertrophic Cardiomyopathy: Clinical Models Still over Genetics

SCD risk stratification in HCM is primarily based on clinical and imaging markers, though predictive accuracy remains limited [46]. Evidence for ICD implantation derives largely from observational studies, and while sarcomeric variants (e.g., MYBPC3, MYH7) are associated with adverse outcomes, their prognostic utility is inconsistent [47]. Current assessment emphasizes phenotypic features (LV wall thickness, syncope, family history of SCD, nonsustained VT, and CMR fibrosis) summarized in the ESC HCM Risk-SCD model, which provides validated 5-year risk estimates in adults [46,47,48]. Pediatric-specific tools offer age-appropriate prediction [2,49], while the role of LGE continues to evolve [50]. ESC guidelines endorse these models, whereas AHA/ACC considers them supportive only [51]. Clinical decision-making must also weigh comorbidities, psychosocial impact, and device-related complications [46]. NSVT, present in up to 25% of patients, is more prognostic in those <30 years [2]. Risk models are not applicable in athletes, syndromic/infiltrative HCM, or peri-myectomy; residual LVOTO and severe hypertrophy may increase risk post-ablation, though evidence remains limited [46].

From a genetic standpoint, MYBPC3 truncating variants (frameshift, nonsense, splice-site) cause nonsense-mediated decay and haploinsufficiency of cardiac myosin-binding protein C, accounting for >40% of genotype-positive HCM. They usually confer later-onset but progressive disease [47]. By contrast, MYH7 missense variants exert dominant-negative effects on β-myosin heavy chain, driving earlier onset and higher arrhythmic risk [52]. Less common variants in TNNT2 and TNNI3 (cardiac troponin T, regulating actin-myosin interaction, and cardiac troponin I, inhibitory subunit of the troponin complex, respectively) may predispose to malignant arrhythmias even in the absence of marked hypertrophy [52]. Tools such as the Toronto genotype score integrate clinical and echocardiographic variables to estimate mutation probability, but are not yet validated for risk prediction [53]. Overall, variant type, location, and pathogenicity may refine SCD risk assessment, but genotype currently complements rather than replaces established phenotypic predictors in guiding ICD eligibility.

### 5.4. Arrhythmogenic Cardiomyopathy: Limitations and Advances

Data on ACM risk stratification remain largely derived from small retrospective cohorts. The annual incidence of VAs ranges from 3.7% to 10.6%, with male sex, right ventricular dysfunction, and prior VT/VF representing the main clinical predictors [54]. Risk models have evolved from the 2015 Task Force algorithm [55] to contemporary AHA/ACC/HRS and HRS guidelines [56,57], and a validated multicenter calculator integrating clinical, ECG, and imaging variables, most accurate in PKP2 carriers, though tending to overestimate risk at low thresholds [58,59]. Clinical judgment and shared decision-making therefore remain essential (Table 1), while fast VT (>250 bpm) is under evaluation as a surrogate for SCD [2].

Genotype-specific data further refine stratification. The TMEM43 c.1073C>T; p.Ser358Leu missense mutation (substituting serine with leucine at codon 358) is associated with a highly malignant phenotype in which SCD may be the first manifestation; in this setting, ICD prophylaxis can be justified on the basis of genotype alone [20]. Similarly, truncating or pathogenic missense variants in DSP define a left-dominant subtype with extensive fibrosis and high arrhythmic burden [44]. A DSP-specific risk score, recently validated in the “DSP-ERADOS” multicenter cohort, is designed for carriers of P/LP DSP variants and integrates clinical and imaging parameters to guide genotype-informed ICD implantation [60]. Truncating (nonsense, splice-site) and missense variants in PKP2 are the most frequent genetic substrate of ACM and consistently confer increased VA risk, underscoring their pivotal role in risk prediction models and ICD eligibility [61]. EPS may still assist in selected symptomatic cases [2].

### 5.5. Restrictive Cardiomyopathy: Managing Heterogeneity

Restrictive cardiomyopathy carries the worst prognosis among cardiomyopathies, with mortality primarily driven by restrictive hemodynamics; in pediatric cohorts, over 50% progress to death or transplantation shortly after diagnosis [62]. Risk stratification for ICD remains challenging due to etiological heterogeneity and limited predictive tools, relying mainly on clinical severity, HF phenotype, and arrhythmic burden [22].

Genetic data increasingly inform this process. Variants in TNNI3 represent the prototypical genetic substrate of primary RCM: most are missense changes that increase Ca^2+^ sensitivity of troponin I, impair relaxation, and produce marked diastolic stiffness with atrial enlargement [63]. Less commonly, TNNT2 mutations can also induce restrictive physiology through defective thin filament regulation, while DES variants (often splice-site or missense) disrupt intermediate filament assembly and predispose to conduction abnormalities [63]. FLNC truncating variants destabilize the Z-disc and typically manifest with a mixed restrictive–arrhythmic phenotype [22,37].

Beyond primary genetic forms, non-primary phenocopies must be considered [22]. Anderson–Fabry disease (X-linked GLA, α-galactosidase A deficiency with glycosphingolipid storage) and hereditary transthyretin amyloidosis (ATTRv, TTR variants causing protein misfolding and deposition) both manifest restrictive physiology but stem from infiltrative rather than sarcomeric mechanisms, with distinct prognostic and therapeutic implications. In Fabry disease, systematic reviews indicate that SCD risk is primarily driven by VAs [64], whereas in cardiac amyloidosis small series have shown limited benefit of prophylactic ICD implantation [65]. Overall, sudden death risk is usually outweighed by progressive heart failure, restricting ICD indication to patients with unstable VAs [2].

**Table 1:** Genetic Basis and Risk Stratification in Cardiomyopathies. Overview of genetic findings across five cardiomyopathy subtypes (DCM, NDLVC, HCM, ACM, RCM), highlighting prevalence of P/LP variants, key genes, and the role of genetic testing in guiding ICD placement for primary prevention of sudden cardiac death. References in brackets correspond to those in the manuscript.

Cardiomyopathy Subtype	Key Genes/Variants	Risk Features	Risk Tools/ICD Implications
Dilated cardiomyopathy (DCM)	LMNA truncating (nonsense/frameshift) → high arrhythmic risk; some missense (exons 7–12) VUS [32,33,34]. PLN p.Arg14del (in-frame deletion) → malignant VAs, risk model validated [35,36]. FLNC truncating → VAs, remodeling; some missense alter splicing/aggregation [37,38,39]. TTN truncating (frameshift, nonsense, splice-site) → common, milder arrhythmic profile, recovery possible [40,41]. BAG3 p.Pro209Leu (missense) → early DCM, HF progression, VAs [23]. TAZ loss-of-function (frameshift, nonsense, splice-site, insertion/deletion) → Barth syndrome [16]. SCN5A missense → arrhythmic DCM [15].	High risk irrespective of LVEF for LMNA, PLN, FLNC; TTN more favorable; BAG3 early HF; TAZ syndromic.	LMNA-risk VTA score [34]; PLN risk score [35]; genotype informs ICD beyond LVEF.
Non-dilated LV cardiomyopathy (NDLVC)	FLNC (truncating) → fibrosis, VAs [37]. DSP truncating → left-dominant ACM; missense in binding domains [44]. PLN p.Arg14del → VAs before dilation [36]. RBM20 missense (RS domain) → aggressive, early-onset, male predominance [45]. TMEM43 p.S358L → highly malignant [20]. DES (missense, splice-site) → fibrosis, arrhythmias [2].	High VA/SCD risk, often with preserved LVEF; subepicardial LGE; male sex increases risk (RBM20).	PLN risk score [35]; ICD if NSVT, family SCD, or LGE (ESC 2023) [2].
Hypertrophic cardiomyopathy (HCM)	MYBPC3 truncating (frameshift, nonsense, splice-site) → haploinsufficiency, later onset [47]. MYH7 missense (dominant-negative) → early, malignant [47,52]. TNNT2, TNNI3 (missense) → malignant arrhythmias without severe hypertrophy [52].	MYBPC3: later/progressive; MYH7: early, higher arrhythmic risk; troponins: malignant arrhythmias.	Risk-SCD model [48]; HCM Risk-Kids [49]; PRIMaCY [2]; LGE role evolving [50].
Arrhythmogenic cardiomyopathy (ACM)	PKP2 (truncating/missense) → frequent ARVC substrate [61]. DSP (truncating/missense) → left-dominant with fibrosis [44,60]. TMEM43 p.S358L (missense) → highly malignant, SCD first event [20]. PLN p.Arg14del founder mutation [21]. DES, JUP, DSG2, DSC2 [2].	PKP2 frequent; DSP left-dominant subtype; TMEM43 malignant; PLN arrhythmogenic.	ARVC Risk Calculator [58,59]; DSP risk score [60]; ICD justified on genotype (e.g., TMEM43).
Restrictive cardiomyopathy (RCM)	TNNI3 (missense) → ↑ Ca^2+^ sensitivity, diastolic stiffness [63]. TNNT2 (missense) → restrictive physiology [63]. FLNC (truncating) → restrictive–arrhythmic [22,37]. DES (missense/splice-site) → conduction abnormalities [63]. BAG3 (incl. p.Pro209Leu) → myofibrillar myopathy [23]. Sarcomeric (MYH7, MYBPC3, ACTC1, TPM1, MYPN, MYL2/3) [2]. GLA loss-of-function (Fabry) [64]; TTR missense (ATTRv) [65].	High mortality; >50% pediatric to Tx/death [62]. Fabry → VA risk; ATTRv → HF-driven mortality.	ICD only for unstable VAs; limited role in amyloidosis [65]; Fabry with VA risk [64].

Table note: AD, autosomal dominant; DCM, dilated cardiomyopathy; NDLVC, non-dilated left ventricular cardiomyopathy; HCM, hypertrophic cardiomyopathy; ACM, arrhythmogenic cardiomyopathy; RCM, restrictive cardiomyopathy; P/LP, pathogenic/likely pathogenic; ICD, implantable cardioverter defibrillator; VAs, ventricular arrhythmias; SCD, sudden cardiac death; LVEF, left ventricular ejection fraction; LGE, late gadolinium enhancement; Tx, transplant.

## 6. Feasibility and Challenges of Genotype-Based ICD Trials

The role of ICD in primary prevention of SCD was initially established by RCTs showing reductions in arrhythmic and all-cause mortality among patients with systolic dysfunction [66,67,68,69]. More recent evidence has refined this paradigm: in non-ischemic cardiomyopathy, the DANISH trial confirmed protection against sudden arrhythmic death but not overall survival [70], with post hoc analyses suggesting benefit largely confined to younger patients [71]. Collectively, these findings have underscored the need for more individualized strategies, integrating etiological context and patient age into risk stratification.

In parallel, recognition of genotype-specific risk has stimulated growing interest in genetics-guided allocation of ICD therapy. P/LP variants affecting nuclear integrity, cytoskeletal structure, calcium handling, or intercellular adhesion are associated with highly arrhythmogenic phenotypes, particularly in DCM and ACM, whereas in HCM clinical and imaging markers remain predominant [2,47]. Accurate interpretation of variants is essential to avoid misclassification; pathogenic variants can meaningfully inform risk, but VUS should not guide clinical decision-making [14]. Observational registries have confirmed the feasibility of integrating genetic information into ICD allocation [32,35], although randomized evidence is still lacking (Supplementary Table S1).

Given the rarity and heterogeneity of high-risk variants, pragmatic alternatives such as prospective genotype registries, multimodal prediction models, and artificial intelligence–based tools are increasingly being explored [58,72]. Looking ahead, gene-based strategies, including allele-specific silencing and CRISPR-based genome editing, may eventually shift prevention from arrhythmia suppression to causal treatment of monogenic cardiomyopathies, though efficacy and safety remain under active investigation [73,74] (Table 1).

## 7. Conclusions

The integration of genotype-informed stratification into clinical pathways marks a paradigm shift in inherited cardiomyopathy management, enabling more precise, personalized, and cost-effective use of ICD therapy.

While a significant residual risk of SCD persists despite optimal GDMT [75], genotype-based models, particularly for high-risk variants, allow for refined risk stratification beyond conventional metrics like LVEF, whereas low-risk genotypes may warrant more conservative strategies [13]. As ongoing trials and registries increasingly integrate genetic, imaging, and clinical data to support individualized decision-making [14], ICD use should shift toward dynamic, genotype-informed models to optimize risk–benefit balance and resource allocation [76,77,78].

## Data Availability

No new data were created or analyzed in this study. Data sharing is not applicable to this article.

## Supplementary Materials

The following supporting information can be downloaded at: https://www.mdpi.com/article/10.3390/biomedicines13112626/s1, Table S1. Qualitative Evaluation of Major Genetic Studies and Risk Scores. Qualitative evaluation of major genetic studies and risk scores. The table summarizes population characteristics, methodological approaches, main outcomes, and implications for ICD allocation across different inherited cardiomyopathies.

## Abbreviations

The following abbreviations are used in this manuscript:

ACM	Arrhythmogenic cardiomyopathy
ARVC	Arrhythmogenic right ventricular cardiomyopathy
CMR	Cardiovascular magnetic resonance
DCM	Dilated cardiomyopathy
EPS	Electrophysiological study
GDMT	Guideline-directed medical therapy
HCM	Hypertrophic cardiomyopathy
HNDCM	Hypokinetic non-dilated cardiomyopathy
ICD	Implantable cardioverter defibrillator
LGE	Late gadolinium enhancement
LV	Left ventricle/Left ventricular
LVEF	Left ventricular ejection fraction
LVOTO	Left ventricle outflow tract obstruction
NDLVC	Non-dilated left ventricular cardiomyopathy
NSVT	Non-sustained ventricular tachycardia
P/LP	Pathogenic/likely pathogenic
RCM	Restrictive cardiomyopathy
RCT(s)	Randomized control trial(s)
RV	Right ventricle/Right ventricular
SCD	Sudden cardiac death
VA(s)	Ventricular arrhythmia(s)
VF	Ventricular fibrillation
VT	Ventricular tachycardia
VUS	Variants of uncertain significance

## References

[1] Corrado D., Link M.S., Schwartz P.J. (2022). Implantable defibrillators in primary prevention of genetic arrhythmias. A shocking choice?. Eur. Heart J..

[2] Arbelo E., Protonotarios A., Gimeno J.R., Arbustini E., Barriales-Villa R., Basso C., Bezzina C.R., Biagini E., Blom N.A., de Boer R.A. (2023). 2023 ESC Guidelines for the management of cardiomyopathies: Developed by the task force on the management of cardiomyopathies of the European Society of Cardiology (ESC). Eur. Heart J..

[3] Zeppenfeld K., Tfelt-Hansen J., de Riva M., Winkel B.G., Behr E.R., Blom N.A., Charron P., Corrado D., Dagres N., de Chillou C. (2022). 2022 ESC Guidelines for the management of patients with ventricular arrhythmias and the prevention of sudden cardiac death: Developed by the task force for the management of patients with ventricular arrhythmias and the prevention of sudden cardiac death of the European Society of Cardiology (ESC) Endorsed by the Association for European Paediatric and Congenital Cardiology (AEPC). Eur. Heart J..

[4] Basso C., Thiene G., Corrado D., Buja G., Melacini P., Nava A. (2000). Hypertrophic cardiomyopathy and sudden death in the young: Pathologic evidence of myocardial ischemia. Hum. Pathol..

[5] Thiene G., Nava A., Corrado D., Rossi L., Pennelli N. (1988). Right ventricular cardiomyopathy and sudden death in young people. N. Engl. J. Med..

[6] Schwartz P.J., Ackerman M.J., Antzelevitch C., Bezzina C.R., Borggrefe M., Cuneo B.F., Wilde A.A.M. (2020). Inherited cardiac arrhythmias. Nat. Rev. Dis. Prim..

[7] Corrado D., Zorzi A., Cerrone M., Rigato I., Mongillo M., Bauce B., Delmar M. (2016). Relationship Between Arrhythmogenic Right Ventricular Cardiomyopathy and Brugada Syndrome: New Insights from Molecular Biology and Clinical Implications. Circ. Arrhythm. Electrophysiol..

[8] Corrado D., Anastasakis A., Basso C., Bauce B., Blomström-Lundqvist C., Bucciarelli-Ducci C., Cipriani A., De Asmundis C., Gandjbakhch E., Jiménez-Jáimez J. (2024). Proposed diagnostic criteria for arrhythmogenic cardiomyopathy: European Task Force consensus report. Int. J. Cardiol..

[9] Olde Nordkamp L.R.A., Postema P.G., Knops R.E., van Dijk N., Limpens J., Wilde A.A.M., de Groot J.R. (2016). Implantable cardioverter-defibrillator harm in young patients with inherited arrhythmia syndromes: A systematic review and meta-analysis of inappropriate shocks and complications. Heart Rhythm.

[10] Olde Nordkamp L.R.A., Wilde A.A.M., Tijssen J.G.P., Knops R.E., van Dessel P.F.H.M., de Groot J.R. (2013). The ICD for primary prevention in patients with inherited cardiac diseases: Indications, use, and outcome: A comparison with secondary prevention. Circ. Arrhythm. Electrophysiol..

[11] Brouwer T.F., Yilmaz D., Lindeboom R., Buiten M.S., Olde Nordkamp L.R.A., Schalij M.J., Wilde A.A., van Erven L., Knops R.E. (2016). Long-Term Clinical Outcomes of Subcutaneous Versus Transvenous Implantable Defibrillator Therapy. J. Am. Coll. Cardiol..

[12] Bettin M., Larbig R., Rath B., Fischer A., Frommeyer G., Reinke F., Köbe J., Eckardt L. (2017). Long-Term Experience with the Subcutaneous Implantable Cardioverter-Defibrillator in Teenagers and Young Adults. JACC Clin. Electrophysiol..

[13] Paldino A., Dal Ferro M., Stolfo D., Gandin I., Medo K., Graw S., Gigli M., Gagno G., Zaffalon D., Castrichini M. (2022). Prognostic Prediction of Genotype vs Phenotype in Genetic Cardiomyopathies. J. Am. Coll. Cardiol..

[14] Burke M.A., Cook S.A., Seidman J.G., Seidman C.E. (2016). Clinical and Mechanistic Insights into the Genetics of Cardiomyopathy. J. Am. Coll. Cardiol..

[15] McNair W.P., Sinagra G., Taylor M.R.G., Di Lenarda A., Ferguson D.A., Salcedo E.E., Slavov D., Zhu X., Caldwell J.H., Mestroni L. (2011). SCN5A mutations associate with arrhythmic dilated cardiomyopathy and commonly localize to the voltage-sensing mechanism. J. Am. Coll. Cardiol..

[16] Schlame M., Kelley R.I., Feigenbaum A., Towbin J.A., Heerdt P.M., Schieble T., Wanders R.J.A., DiMauro S., Blanck T.J.J. (2003). Phospholipid abnormalities in children with Barth syndrome. J. Am. Coll. Cardiol..

[17] Escobar-López L., Ochoa J.P., Mirelis J.G., Espinosa M.Á., Navarro M., Gallego-Delgado M., Barriales-Villa R., Robles-Mezcua A., Basurte-Elorz M.T., Gutiérrez García-Moreno L. (2021). Association of Genetic Variants with Outcomes in Patients with Nonischemic Dilated Cardiomyopathy. J. Am. Coll. Cardiol..

[18] Pelliccia A., Lemme E., Maestrini V., Di Paolo F.M., Pisicchio C., Di Gioia G., Caselli S. (2018). Does Sport Participation Worsen the Clinical Course of Hypertrophic Cardiomyopathy?. Circulation.

[19] Marcus F.I., McKenna W.J., Sherrill D., Basso C., Bauce B., Bluemke D.A., Calkins H., Corrado D., Cox M.G.P.J., Daubert J.P. (2010). Diagnosis of arrhythmogenic right ventricular cardiomyopathy/Dysplasia: Proposed modification of the task force criteria. Circulation.

[20] Hodgkinson K.A., Howes A.J., Boland P., Shen X.S., Stuckless S., Young T.-L., Curtis F., Collier A., Parfrey P.S., Connors S.P. (2016). Long-Term Clinical Outcome of Arrhythmogenic Right Ventricular Cardiomyopathy in Individuals with a p.S358L Mutation in TMEM43 Following Implantable Cardioverter Defibrillator Therapy. Circ. Arrhythmia Electrophysiol..

[21] van der Zwaag P.A., van Rijsingen I.A.W., Asimaki A., Jongbloed J.D.H., van Veldhuisen D.J., Wiesfeld A.C.P., Cox M.G.P.J., van Lochem L.T., de Boer R.A., Hofstra R.M.W. (2012). Phospholamban R14del mutation in patients diagnosed with dilated cardiomyopathy or arrhythmogenic right ventricular cardiomyopathy: Evidence supporting the concept of arrhythmogenic cardiomyopathy. Eur. J. Heart Fail..

[22] Rapezzi C., Aimo A., Barison A., Emdin M., Porcari A., Linhart A., Keren A., Merlo M., Sinagra G. (2022). Restrictive cardiomyopathy: Definition and diagnosis. Eur. Heart J..

[23] Meister-Broekema M., Freilich R., Jagadeesan C., Rauch J.N., Bengoechea R., Motley W.W., Kuiper E.F.E., Minoia M., Furtado G.V., van Waarde M.A.W.H. (2018). Myopathy-associated BAG3 mutations lead to protein aggregation by stalling Hsp70 networks. Nat. Commun..

[24] Lucas Laws J., Lancaster M.C., Shoemaker M.B., Stevenson W.G., Hung R.R., Wells Q., Brinkley D.M., Hughes S., Anderson K., Roden D. (2022). Arrhythmias as Presentation of Genetic Cardiomyopathy. Circ. Res..

[25] Sato P.Y., Musa H., Coombs W., Guerrero-Serna G., Patiño G.A., Taffet S.M., Isom L.L., Delmar M. (2009). Loss of plakophilin-2 expression leads to decreased sodium current and slower conduction velocity in cultured cardiac myocytes. Circ. Res..

[26] Watanabe H., Yang T., Stroud D.M., Lowe J.S., Harris L., Atack T.C., Wang D.W., Hipkens S.B., Leake B., Hall L. (2011). Striking in vivo phenotype of a disease-associated human scn5a mutation producing minimal changes in vitro. Circulation.

[27] Raymond-Paquin A., Nattel S., Wakili R., Tadros R. (2018). Mechanisms and Clinical Significance of Arrhythmia-Induced Cardiomyopathy. Can. J. Cardiol..

[28] Hookana E., Junttila M.J., Puurunen V.-P., Tikkanen J.T., Kaikkonen K.S., Kortelainen M.-L., Myerburg R.J., Huikuri H.V. (2011). Causes of nonischemic sudden cardiac death in the current era. Heart Rhythm.

[29] Junttila M.J., Holmström L., Pylkäs K., Mantere T., Kaikkonen K., Porvari K., Kortelainen M.-L., Pakanen L., Kerkelä R., Myerburg R.J. (2018). Primary Myocardial Fibrosis as an Alternative Phenotype Pathway of Inherited Cardiac Structural Disorders. Circulation.

[30] Gupta S., Ge Y., Singh A., Gräni C., Kwong R.Y. (2021). Multimodality Imaging Assessment of Myocardial Fibrosis. JACC Cardiovasc. Imaging.

[31] Alba A.C., Foroutan F., Duero Posada J., Battioni L., Schofield T., Alhussein M., Agoritsas T., Spencer F.A., Guyatt G. (2018). Implantable cardiac defibrillator and mortality in non-ischaemic cardiomyopathy: An updated meta-analysis. Heart.

[32] Wahbi K., Ben Yaou R., Gandjbakhch E., Anselme F., Gossios T., Lakdawala N.K., Stalens C., Sacher F., Babuty D., Trochu J.-N. (2019). Development and Validation of a New Risk Prediction Score for Life-Threatening Ventricular Tachyarrhythmias in Laminopathies. Circulation.

[33] Bhaskaran A., Ben Yaou R., Helms A.S., Fayssoil A., Richard P., Stojkovic T., Anselme F., Labombarda F., Chikhaoui C., De Sandre-Giovannoni A. (2025). Location of LMNA variants and clinical outcomes in cardiomyopathy. JAMA Cardiol..

[34] Rootwelt-Norberg C., Christensen A.H., Skjølsvik E.T., Chivulescu M., Vissing C.R., Bundgaard H., Aabel E.W., Bogsrud M.P., Hasselberg N.E., Lie Ø.H. (2023). Timing of cardioverter-defibrillator implantation in patients with cardiac laminopathies—External validation of the LMNA-risk ventricular tachyarrhythmia calculator. Heart Rhythm.

[35] Verstraelen T.E., Van Lint F.H.M., Bosman L.P., De Brouwer R., Proost V.M., Abeln B.G.S., Taha K., Zwinderman A.H., Dickhoff C., Oomen T. (2021). Prediction of ventricular arrhythmia in phospholamban p.Arg14del mutation carriers-reaching the frontiers of individual risk prediction. Eur. Heart J..

[36] Vafiadaki E., Chaudhari I., Soliman K.M., Eliopoulos A.G., Kranias E.G., Sanoudou D. (2025). Genetic landscape of phospholamban cardiomyopathies. Front. Cell Dev. Biol..

[37] Gigli M., Stolfo D., Graw S.L., Merlo M., Gregorio C., Nee Chen S., Dal Ferro M., Paldino A., De Angelis G., Brun F. (2021). Phenotypic Expression, Natural History, and Risk Stratification of Cardiomyopathy Caused by Filamin C Truncating Variants. Circulation.

[38] Akhtar M.M., Lorenzini M., Pavlou M., Ochoa J.P., O’Mahony C., Restrepo-Cordoba M.A., Segura-Rodriguez D., Bermúdez-Jiménez F., Molina P., Cuenca S. (2021). Association of Left Ventricular Systolic Dysfunction Among Carriers of Truncating Variants in Filamin C with Frequent Ventricular Arrhythmia and End-stage Heart Failure. JAMA Cardiol..

[39] Roldán-Sevilla T., Pérez-Serra A., Campuzano O., Sarquella-Brugada G., Cesar S., Arbelo E. (2019). Missense mutations in the FLNC gene causing familial dilated cardiomyopathy. Circ. Genom. Precis. Med..

[40] van der Zwaag P.A., Van Rijsingen I.A.W., De Ruiter R., Nannenberg E.A., Groeneweg J.A., Post J.G., Hauer R.N.W., Van Gelder I.C., Van den Berg M.P., Van der Harst P. (2013). Recurrent and founder mutations in the Netherlands: RBM20 mutations in cardiomyopathy. Neth. Heart J..

[41] Herman D.S., Lam L., Taylor M.R.G., Wang L., Teekakirikul P., Christodoulou D., Conner L., DePalma S.R., McDonough B., Sparks E. (2012). Truncations of titin causing dilated cardiomyopathy. N. Engl. J. Med..

[42] Pontone G., Guaricci A.I., Andreini D., Solbiati A., Guglielmo M., Mushtaq S., Baggiano A., Beltrama V., Fusini L., Rota C. (2016). Prognostic Benefit of Cardiac Magnetic Resonance over Transthoracic Echocardiography for the Assessment of Ischemic and Nonischemic Dilated Cardiomyopathy Patients Referred for the Evaluation of Primary Prevention Implantable Cardioverter-Defibrillator Therapy. Circ. Cardiovasc. Imaging.

[43] Klem I., Klein M., Khan M., Yang E.Y., Nabi F., Ivanov A., Bhatti L., Hayes B., Graviss E.A., Nguyen D.T. (2021). Relationship of LVEF and Myocardial Scar to Long-Term Mortality Risk and Mode of Death in Patients with Nonischemic Cardiomyopathy. Circulation.

[44] Smith E.D., Lakdawala N.K., Papoutsidakis N., Aubert G., Mazzanti A., McCanta A.C., Agarwal P.P., Arscott P., Dellefave-Castillo L.M., Vorovich E.E. (2020). Desmoplakin cardiomyopathy, a novel phenotype of arrhythmogenic left ventricular cardiomyopathy: Natural history and genotype-phenotype correlations. Heart Rhythm.

[45] van den Hoogenhof M.M.G., Beqqali A., Amin A.S., van der Made I., Aufiero S., Khan M.A.F., Schumacher C.A., Jansweijer J.A., van Spaendonck-Zwarts K.Y., Remme C.A. (2018). RBM20 mutations induce an arrhythmogenic dilated cardiomyopathy related to disturbed calcium handling. Circulation.

[46] Spirito P., Bellone P., Harris K.M., Bernabò P., Bruzzi P., Maron B.J. (2000). Magnitude of left ventricular hypertrophy and risk of sudden death in hypertrophic cardiomyopathy. N. Engl. J. Med..

[47] Ho C.Y., Day S.M., Ashley E.A., Michels M., Pereira A.C., Jacoby D., Cirino A.L., Fox J.C., Lakdawala N.K., Ware J.S. (2018). Genotype and lifetime burden of disease in hypertrophic cardiomyopathy insights from the sarcomeric human cardiomyopathy registry (SHaRe). Circulation.

[48] O’Mahony C., Jichi F., Pavlou M., Monserrat L., Anastasakis A., Rapezzi C., Biagini E., Gimeno J.R., Limongelli G., McKenna W.J. (2014). A novel clinical risk prediction model for sudden cardiac death in hypertrophic cardiomyopathy (HCM Risk-SCD). Eur. Heart J..

[49] Norrish G., Ding T., Field E., Ziółkowska L., Olivotto I., Limongelli G., Anastasakis A., Weintraub R., Biagini E., Ragni L. (2019). Development of a Novel Risk Prediction Model for Sudden Cardiac Death in Childhood Hypertrophic Cardiomyopathy (HCM Risk-Kids). JAMA Cardiol..

[50] Pan J.A. (2024). Late Gadolinium Enhancement in Hypertrophic Cardiomyopathy: Is There More to It Than Size?. JACC Cardiovasc. Imaging..

[51] Leggit J.C., Whitaker D. (2022). Diagnosis and Management of Hypertrophic Cardiomyopathy: Updated Guidelines From the ACC/AHA. Am. Fam. Physician.

[52] Richard P., Charron P., Carrier L., Ledeuil C., Cheav T., Pichereau C., Benaiche A., Isnard R., Dubourg O., Burban M. (2003). Hypertrophic cardiomyopathy: Distribution of disease genes, spectrum of mutations, and implications for a molecular diagnosis strategy. Circulation.

[53] Gruner C., Ivanov J., Care M., Williams L., Moravsky G., Yang H., Laczay B., Siminovitch K., Woo A., Rakowski H. (2013). Toronto Hypertrophic Cardiomyopathy Genotype Score for Prediction of a Positive Genotype in Hypertrophic Cardiomyopathy. Circ. Cardiovasc. Genet..

[54] Bosman L.P., Sammani A., James C.A., Cadrin-Tourigny J., Calkins H., van Tintelen J.P., Hauer R.N.W., Asselbergs F.W., te Riele A.S.J.M. (2018). Predicting arrhythmic risk in arrhythmogenic right ventricular cardiomyopathy: A systematic review and meta-analysis. Heart Rhythm.

[55] Corrado D., Wichter T., Link M.S., Hauer R.N.W., Marchlinski F.E., Anastasaki A., Bauce B., Basso C., Brunckhorst C., Tsatsopoulou A. (2015). Treatment of arrhythmogenic right ventricular cardiomyopathy/dysplasia: An international task force consensus statement. Eur. Heart J..

[56] Al-Khatib S.M., Stevenson W.G., Ackerman M.J., Bryant W.J., Callans D.J., Curtis A.B., Deal B.J., Dickfeld T., Field M.E., Fonarow G.C. (2018). 2017 AHA/ACC/HRS Guideline for Management of Patients With Ventricular Arrhythmias and the Prevention of Sudden Cardiac Death: A Report of the American College of Cardiology/American Heart Association Task Force on Clinical Practice Guidelines and the Hea. J. Am. Coll. Cardiol..

[57] Towbin J.A., McKenna W.J., Abrams D.J., Ackerman M.J., Calkins H., Darrieux F.C.C., Daubert J.P., de Chillou C., DePasquale E.C., Desai M.Y. (2019). 2019 HRS expert consensus statement on evaluation, risk stratification, and management of arrhythmogenic cardiomyopathy. Heart Rhythm.

[58] Cadrin-Tourigny J., Bosman L.P., Nozza A., Wang W., Tadros R., Bhonsale A., Bourfiss M., Fortier A., Lie Ø.H., Saguner A.M. (2019). A new prediction model for ventricular arrhythmias in arrhythmogenic right ventricular cardiomyopathy. Eur. Heart J..

[59] Protonotarios A., Bariani R., Cappelletto C., Pavlou M., García-García A., Cipriani A., Protonotarios I., Rivas A., Wittenberg R., Graziosi M. (2022). Importance of genotype for risk stratification in arrhythmogenic right ventricular cardiomyopathy using the 2019 ARVC risk calculator. Eur. Heart J..

[60] Carrick R.T., Gasperetti A., Protonotarios A., Murray B., Laredo M., van der Schaaf I., Dooijes D., Syrris P., Cannie D., Tichnell C. (2024). A novel tool for arrhythmic risk stratification in desmoplakin gene variant carriers. Eur. Heart J..

[61] Bhonsale A., Groeneweg J.A., James C.A., Dooijes D., Tichnell C., Jongbloed J.D.H., Murray B., te Riele A.S.J.M., van den Berg M.P., Bikker H. (2015). Impact of genotype on clinical course in arrhythmogenic right ventricular dysplasia/cardiomyopathy-associated mutation carriers. Eur. Heart J..

[62] Muchtar E., Blauwet L.A., Gertz M.A. (2017). Restrictive cardiomyopathy: Genetics, pathogenesis, clinical manifestations, diagnosis, and therapy. Circ. Res..

[63] Yang Z., Chen J., Li H., Lin Y. (2022). Genotype-Phenotype Associations with Restrictive Cardiomyopathy Induced by Pathogenic Genetic Mutations. Rev. Cardiovasc. Med..

[64] Baig S., Edward N.C., Kotecha D., Liu B., Nordin S., Kozor R., Moon J.C., Geberhiwot T., Steeds R.P. (2018). Ventricular arrhythmia and sudden cardiac death in Fabry disease: A systematic review of risk factors in clinical practice. EP Eur..

[65] Kristen A.V., Dengler T.J., Hegenbart U., Schonland S.O., Goldschmidt H., Sack F.-U., Voss F., Becker R., Katus H.A., Bauer A. (2008). Prophylactic implantation of cardioverter-defibrillator in patients with severe cardiac amyloidosis and high risk for sudden cardiac death. Heart Rhythm.

[66] Moss A.J., Hall W.J., Cannom D.S., Daubert J.P., Higgins S.L., Klein H., Levine J.H., Saksena S., Waldo A.L., Wilber D. (1996). Improved Survival with an Implanted Defibrillator in Patients with Coronary Disease at High Risk for Ventricular Arrhythmia. N. Engl. J. Med..

[67] Moss A.J., Zareba W., Hall W.J., Klein H., Wilber D.J., Cannom D.S., Daubert J.P., Higgins S.L., Brown M.W., Andrews M.L. (2002). Prophylactic Implantation of a Defibrillator in Patients with Myocardial Infarction and Reduced Ejection Fraction. N. Engl. J. Med..

[68] Bardy G.H., Lee K.L., Mark D.B., Poole J.E., Packer D.L., Boineau R., Domanski M., Troutman C., Anderson J., Johnson G. (2005). Amiodarone or an Implantable Cardioverter-Defibrillator for Congestive Heart Failure. N. Engl. J. Med..

[69] Kadish A., Dyer A., Daubert J.P., Quigg R., Estes N.A.M., Anderson K.P., Calkins H., Hoch D., Goldberger J., Shalaby A. (2004). Prophylactic Defibrillator Implantation in Patients with Nonischemic Dilated Cardiomyopathy. N. Engl. J. Med..

[70] Køber L., Thune J.J., Nielsen J.C., Haarbo J., Videbæk L., Korup E., Jensen G., Hildebrandt P., Steffensen F.H., Bruun N.E. (2016). Defibrillator Implantation in Patients with Nonischemic Systolic Heart Failure. N. Engl. J. Med..

[71] Elming M.B., Nielsen J.C., Haarbo J., Videbæk L., Korup E., Signorovitch J., Olesen L.L., Hildebrandt P., Steffensen F.H., Bruun N.E. (2017). Age and outcomes of primary prevention implantable cardioverter-defibrillators in patients with nonischemic systolic heart failure. Circulation.

[72] Kolk M.Z.H., Ruipérez-Campillo S., Wilde A.A.M., Knops R.E., Narayan S.M., Tjong F.V.Y. (2025). Prediction of sudden cardiac death using artificial intelligence: Current status and future directions. Heart Rhythm.

[73] Kim Y., Landstrom A.P., Shah S.H., Wu J.C., Seidman C.E. (2024). Gene Therapy in Cardiovascular Disease: Recent Advances and Future Directions in Science: A Science Advisory from the American Heart Association. Circulation.

[74] Van Linthout S., Stellos K., Giacca M., Bertero E., Cannata A., Carrier L., Garcia-Pavia P., Ghigo A., González A., Haugaa K.H. (2025). State of the art and perspectives of gene therapy in heart failure. A scientific statement of the Heart Failure Association of the ESC, the ESC Council on Cardiovascular Genomics and the ESC Working Group on Myocardial & Pericardial Diseases. Eur. J. Heart Fail..

[75] Leyva F., Israel C.W., Singh J. (2023). Declining Risk of Sudden Cardiac Death in Heart Failure: Fact or Myth?. Circulation.

[76] Study of Personalized Allocation of Defibrillators in Non-Ischemic Heart Failure (SPANISH-1). ClinicalTrials.gov Identifier: NCT06055504. Sponsor: Consorcio Centro de Investigación Biomédica en Red (CIBER). Status: Active—Recruiting. NCT06055504.

[77] Selvanayagam J.B., Hartshorne T., Billot L., Grover S., Hillis G.S., Jung W., Krum H., Prasad S., McGavigan A.D. (2017). Cardiovascular Magnetic Resonance-GUIDEd management of mild to moderate left ventricular systolic dysfunction (CMR GUIDE): Study protocol for a randomized controlled trial. Ann. Noninvasive Electrocardiol..

[78] Cardiac Magnetic Resonance Guidance of Implantable Cardioverter Defibrillator Implantation in Non-ischaemic Dilated Cardiomyopathy (CMR-ICD-DZHK23). ClinicalTrials.gov (U.S. National Library of Medicine, Bethesda, MD, USA). Identifier: NCT04558723. Sponsor: German Centre for Cardiovascular Research (DZHK). Status: Recruiting. NCT04558723.

