# Supplementary material for: Risk Stratification and Optimal Use of Implantable Cardioverter-Defibrillator Therapy in Primary Prevention of Sudden Cardiac Death in Genetic Cardiomyopathies, with Assessment of the Role of Genetic Variants in Guiding Therapeutic Decisions
Source: Biomedicines. 2025 Oct 27;13(11):2626. doi: 10.3390/biomedicines13112626 (PMC12650546; doi:10.3390/biomedicines13112626)
Supplement: Supplementary file 1 [file biomedicines-13-02626-s001.zip › biomedicines-3824508-supplementary.pdf]

Supplementary Table S1. Qualitative Evaluation of Major Genetic Studies and Risk Scores. Qualitative evaluation of major genetic studies and risk scores. The table summarizes population characteristics, methodological approaches, main outcomes, and implications for ICD allocation across different inherited cardiomyopathies.

Study / Reference	Population & Sample Size	Methods / Comparisons	Key Outcomes	Relevance to ICD Allocation
Van Rijsingen et al., 2012 (LMNA cohort)	LMNA mutation carriers, multicenter European cohort	Observational registry	Predictors: male sex, EF ≤45%, NSVT, conduction defects	Foundation for LMNA-risk score; informs ICD beyond EF
Wahbi et al., 2019; Rootwelt-Norberg et al., 2023; Bhaskaran et al., 2025 (LMNA-risk VTA Score)	LMNA carriers, validation cohorts	Risk score derivation & validation	Integrated truncating variants, EF, NSVT, conduction abnormalities	Individualized ICD timing in laminopathies
Verstraelen et al., 2021; Vafiadaki et al., 2025 (PLN p.Arg14del)	Founder mutation cohorts (n≈200+)	Risk model development & follow-up	High incidence of malignant arrhythmias despite preserved EF	Supports ICD independent of EF
Gigli et al., 2021; Akhtar et al., 2021 (FLNC truncating variants)	DCM/NDLVC patients, n≈300–400	Observational registries	High arrhythmia and HF progression with truncating variants	ICD considered even with preserved/mildly reduced EF
Smith et al., 2020; Carrick et al., 2024 (DSP-specific & DSP-ERADOS Score)	DSP variant carriers, multicenter cohorts	Genotype–phenotype analysis; risk score development	DSP truncating = left-dominant arrhythmogenic phenotype	DSP-specific ICD guidance
Cadrin-Tourigny et al., 2019; Protonotarios et al., 2022 (ARVC Risk Calculator)	ARVC patients, multicenter registries	Prediction models	Improved risk prediction vs. guidelines; genotype influences outcomes	Supports ICD in high-risk ARVC
Ho et al., 2018 (SHaRe Registry, HCM)	Large international HCM cohort	Observational registry	MYH7 = early onset, higher risk; MYBPC3 = later onset	Genetics complements clinical risk models
Kaski et al., 2019 (HCM Risk-Kids); O'Mahony et al., 2014 (HCM Risk-SCD)	Pediatric/adult HCM cohorts	Risk prediction models	Validated clinical scores; genotype not integrated	ICD allocation remains phenotype-driven
Hodgkinson et al., 2016 (TMEM43	Canadian founder	Observational follow-up	Highly malignant phenotype; SCD may	Genotype alone may justify prophylactic ICD

p.S358L)	mutation cohort		be first manifestation	
Baig et al., 2018 (Fabry); Kristen et al., 2008 (Amyloidosis)	Fabry disease and ATTR amyloidosis cohorts	Systematic review / case series	Fabry: arrhythmia-driven SCD; Amyloidosis: HF-driven mortality	ICD selective in Fabry; limited role in amyloidosis

Table note: ICD = implantable cardioverter-defibrillator; EF = ejection fraction; VA = ventricular arrhythmia; NSVT = non-sustained ventricular tachycardia; HCM = hypertrophic cardiomyopathy; DCM = dilated cardiomyopathy; NDLVC = non-dilated left ventricular cardiomyopathy; ARVC = arrhythmogenic right ventricular cardiomyopathy.
